# Effectiveness and safety of nurse-led different epidural analgesia methods based on symptom management theory in patients with severe acute pancreatitis: A single-center RCT protocol

**DOI:** 10.1371/journal.pone.0337803

**Published:** 2026-01-02

**Authors:** You Yuan, Qian Luo, Feiyu Yan, Li Li, Lifeng He, Yan Zhou, Chunlan Wu, Mingli Zhong, Fang Chen, Xia Zhang, Junxi Chen, Bao Fu, Rujun Hu

**Affiliations:** 1 Department of Critical Care Medicine, Affiliated Hospital of Zunyi Medical University, Zunyi, Guizhou, People’s Republic of China; 2 Department of Nursing, Affiliated Hospital of Zunyi Medical University, Zunyi, Guizhou, People’s Republic of China; 3 School of Nursing, Zunyi Medical University, Zunyi, Guizhou, People’s Republic of China; Radboud University Medical Center, NETHERLANDS, KINGDOM OF THE

## Abstract

**Background:**

Patients with severe acute pancreatitis (SAP) often experience intense pain that significantly impacts their quality of life and may hinder recovery. Current pain management approaches have limitations. Although continuous thoracic epidural analgesia (TEA) is generally effective, some patients still need additional intermittent injections of local anesthetics for sufficient pain relief. In the ICU, nurses are the primary providers responsible for pain management. Symptom Management Theory (SMT), though used in various fields, has not yet been fully validated for nurse-led pain management employing different TEA administration methods in SAP.

**Objective:**

This protocol aims to assess the effectiveness and safety of SMT-based nurse-led TEA administration methods in patients with SAP, with the goal of optimizing pain management protocols.

**Methods and analysis:**

This single-center, randomized, controlled clinical trial will enroll 76 patients with SAP, randomized into two groups (N = 38 each). The control group will receive continuous TEA with a 50 ml mixture containing nalbuphine 40 mg, ropivacaine 225 mg, lidocaine 0.2 g, and 6 ml of 0.9% saline, administered at 3–5 ml/h. If the patient’s pain score (VAS-P) exceeds 3 or becomes intolerable, the infusion rate will be increased by 1–2 ml/h. In the experimental group, under SMT-guided nurse-led TEA management, nurses will report pain scores exceeding 3 to physicians, and intermittent epidural injections of 5 ml of the same mixture will be administered according to medical prescription, at intervals of ≥2 hours. Pain responses and patient feedback will be continuously monitored to guide timely adjustments of analgesics.

The primary outcome is VAS-P. Secondary outcomes include intra-abdominal pressure (IAP), total dose of combined medication, number of additional interventions, duration of epidural placement, and incidence of 16 predefined adverse events. Endpoint outcomes consist of puncture success rate, ICU length of stay, 28-day mortality, and patient satisfaction with analgesia.

Data will be analyzed using SPSS. Repeated measures data (e.g., VAS-P and IAP at 0 h, 1 h, 6 h, 12 h, 24 h, 48 h, and 72 h) will be examined with a multivariate mixed-effects repeated measures model (MMRM), which appropriately handles data missing at random. Other secondary outcomes, adverse events, and endpoint measures will be analyzed with descriptive statistics, and between-group comparisons will be conducted using t-tests, Mann-Whitney U tests, χ² tests, or Fisher’s exact tests, as appropriate.

**Expected conclusion:**

The experimental group is anticipated to demonstrate superior pain management outcomes compared to the control group, with no increase in adverse events. The SMT-based nurse-led TEA protocol is expected to be safe and effective in improving analgesia, reducing ICU length of stay, decreasing 28-day mortality, and enhancing patient satisfaction. This study will provide new insights and methods for pain management in SAP patients, thereby promoting optimization of clinical practice.

## Introduction

In patients with severe acute pancreatitis (SAP), pain initially presents as vague discomfort lasting 1–3 days, then advances to intense abdominal pain that can persist for days or weeks, often radiating to the lower back, with a complex pain mechanism [1]. If the pain is not managed promptly and effectively, it can significantly affect the patient’s comfort and emotional well-being and may delay recovery [2,3]. There are limitations to using common opioids and nonsteroidal anti-inflammatory drugs (NSAIDs) in patients with SAP. Opioids may worsen the condition and cause ongoing side effects such as respiratory depression, drowsiness, delirium, myoclonus, nausea, vomiting, constipation, immunosuppression, endocrine disruption, and addiction. NSAIDs can cause gastrointestinal problems [4–6]. Therefore, proper pain control is essential for improving the outlook for patients with SAP. A careful pain management plan can not only reduce pain but also limit drug side effects, enhance quality of life, and support recovery [7]. When developing a pain management plan, the individual patient’s circumstances should be carefully considered, and appropriate medications and treatments selected to achieve the best results.

Current pain management protocols often lack specificity and individualization, failing to address the unique needs of different patients [3,8]. Thoracic epidural anesthesia (TEA) provides reversible control of sympathetic nerve activity by injecting a low concentration of local anesthetic into the epidural space at the thoracic spine level (usually T7 to T9) to block regional nerve roots [9,10]. This type of anesthesia not only delivers adequate analgesia and anti-inflammatory effects but also dilates visceral blood vessels, improves organ perfusion, and significantly reduces complications such as intestinal paralysis in patients with acute pancreatitis [11–13]. Continuous infusion of combined drugs via TEA has been widely used in clinical practice and proves to be an effective pain management method [14,15]. However, clinical experience shows that some patients still experience inadequate pain relief after continuous TEA infusion and require additional intermittent injections of local anesthetics for proper pain control. A small study involving 13 patients with epidural analgesia demonstrated that, over 12 days, the median fluctuation in pain scores ranged from 0.2 to 3 points [9]. These results indicate that a single TEA continuous infusion regimen may not satisfy all patients, and on-demand intermittent epidural injections of local anesthetics could serve as a useful complementary approach. Nonetheless, a gap remains in comparative studies of these two strategies, limiting our overall understanding of pain management in patients with SAP.

The Symptom Management Theory (SMT) has achieved significant success in various fields [16,17]. It helps cancer patients reduce pain and nausea, improving their quality of life; [18,19] in patients with chronic disease, In patients with chronic diseases, symptom management theory interventions can reduce symptoms, burden, anxiety, depression, and the degree of suffering; [20] and in ICU patients with endotracheal intubation, it relieves discomfort such as thirst, enhancing overall experience [21]. Through nurse-led assessments and interventions, SMT plays a vital role in managing these symptoms [22,23]. However, further research is needed in the area of epidural analgesia [24].

In the ICU, pain management is regarded as the “fifth vital sign” [25]. For patients with SAP, controlling pain is especially crucial. Since nurses are the healthcare providers who interact most directly with patients, they can quickly and thoroughly evaluate pain symptoms and often take the lead in pain management [26]. Nurse-led pain management involves a comprehensive understanding of the patient’s pain through both subjective and objective assessments, offering a scientific basis for customized treatment plans [27]. Nurses must actively collaborate with physicians, tailoring interventions based on each patient’s specific condition, using SMT as a framework. They continuously monitor changes in pain, promptly adjust treatment measures, and record outcomes and feedback. This highlights the essential role of nurses in pain management and their irreplaceable contribution [28,29].

Although TEA is widely used in clinical practice, pain management in SAP patients continues to be challenging, mainly because of the limitations of a single TEA regimen. Currently, there is no systematic comparison of different epidural drug delivery methods, especially nurse-led personalized approaches based on SMT. Therefore, this research protocol aims to evaluate the effectiveness and safety of different nurse-led epidural analgesia methods in patients with SAP, offering more precise and personalized pain management options for clinical practice.

## Methods

### Study design

**Fig 1:**
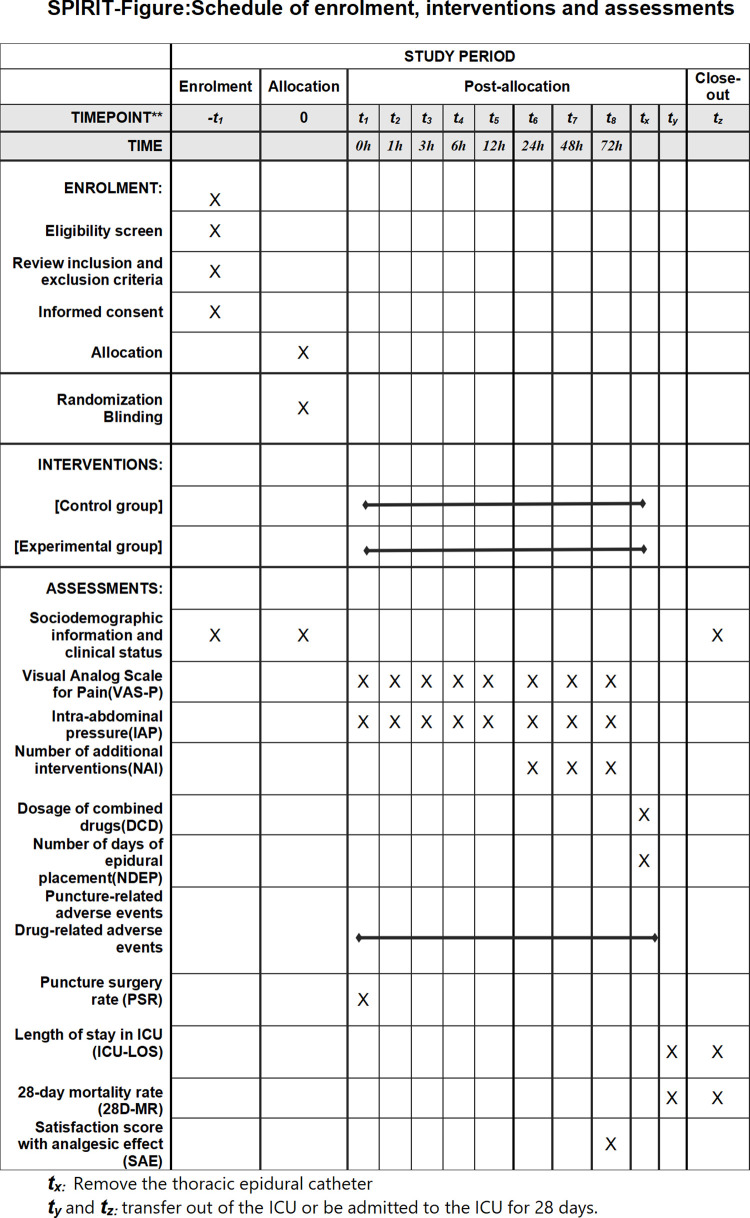
SPIRIT-Figure: Schedule of enrolment, interventions, and assessments.

This protocol is reported in accordance with the Standard Protocol Items: Recommendations for Interventional Trials (SPIRIT) guidelines [30]. The SPIRIT figure, illustrating the schedule of enrolment, interventions, and assessments, is presented in Fig 1, and the SPIRIT checklist is provided in the supplementary S1 File. Study recruitment is planned to commence in June 2025 and is expected to continue until February 2026. The research protocol (Version Number: V1.0; Date: January 1, 2025) and the informed consent form (Version Number: V2.0; Date: February 7, 2025) are key reference documents for this study. Written informed consent will be obtained from each participant or their authorized representative prior to enrolment.

### Ethics, dissemination, and trial registration

Ethics approval was granted by the Biomedical Research Ethics Committee of the Affiliated Hospital of Zunyi Medical University (approval number KLL-2024–695, dated February 10, 2025). The study is registered with the Chinese Clinical Trial Register (ChiCTR) at http://www.chictr.org.cn, under registration number ChiCTR2500101922, with the registration date of May 6, 2025.

### Theory evidence

Based on clinical practice, the team will search domestic and international databases to gather the best evidence-based information and will develop pain management strategies using the SMT theoretical framework. This theory will focus on managing patients’ symptoms, including symptom experience, management strategies, and outcomes. In the ICU, in response to pain in patients with SAP, SMT will provide a structured approach to identifying pain symptoms by regularly assessing pain levels with the VAS-P or patient self-report, recording scores, and understanding the patient’s symptom experience. Based on these assessments, a 6W2H pain symptom management strategy will be developed to personalize symptom care. The plan will include two regimens of continuous epidural infusion of combination medications and on-demand intermittent epidural injections of 5 ml of combination medications.

**Fig 2:**
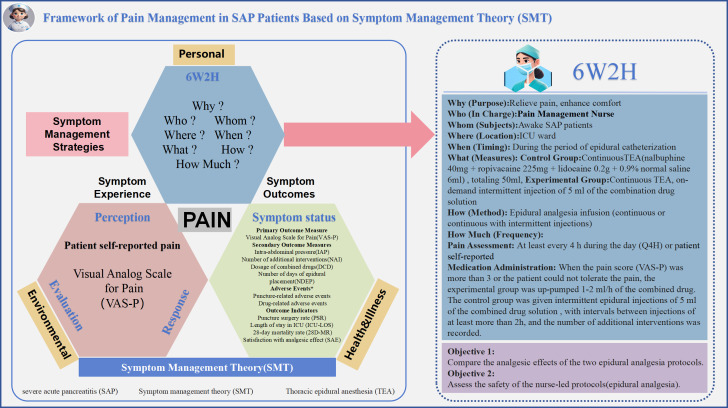
Framework of Pain Management in SAP Patients Based on Symptom Management Theory (SMT).

The key elements—why (purpose), who (in charge), who (subject), where (location), when (timing), what (measures), how (methods), and how much (frequency)—will be specified. Symptom outcomes will refer to the assessment of a symptom management strategy’s effectiveness after implementation. In this study protocol, the effectiveness of pain management will be primarily evaluated through indicators such as pain scores, medication dosages, incidence of adverse events, length of ICU stay, mortality, and patient satisfaction with pain relief. By recording and analyzing these indicators, the study will assess the effectiveness and safety of different analgesic regimens to provide a scientific basis for optimizing pain management strategies. A comprehensive baseline assessment of other factors influencing pain—including person, environment, and health/illness—will be conducted to ensure consistent patient inclusion criteria. The SMT-based pain management framework for SAP patients is presented in Fig 2.

### Study protocol content

This study protocol is a single-center, randomized, controlled clinical trial designed to include 76 patients with SAP, who will be randomly divided into two groups to assess the efficacy and safety of different SMT-based nurse-led epidural analgesia delivery methods. In the control group, continuous TEA will be administered with a combination of nalbuphine 40 mg, ropivacaine 225 mg, lidocaine 0.2 g, and 0.9% saline 6 mL, in a total volume of 50 mL, at a rapid rate of 3–5 mL/h. When the patient’s VAS-P exceeds 3 points or when the patient is unable to tolerate the pain, the infusion rate of the combined solution will be appropriately adjusted upward by 1–2 mL/h.

In the experimental group, based on continuous TEA, a pain management nurse will lead the TEA pain management strategy according to the SMT, reporting to the doctor when the VAS-P exceeds 3 points or when the patient cannot tolerate the pain, and will inject 5 mL of the combined drug solution intermittently, with intervals longer than 2 hours each time, as prescribed by the doctor. The patient’s pain response and feedback will also be continuously monitored to allow for timely adjustments in analgesic measures.

The primary outcome indicator will be VAS-P, while secondary outcome indicators will include intra-abdominal pressure (IAP), number of additional interventions (NAI), dosage of combined drugs (DCD), number of days of epidural placement (NDEP), and 16 adverse events (AEs). Other outcome indicators will include puncture success rate (PSR), length of stay in ICU (ICU-LOS), 28-day mortality rate (28D-MR), and patient satisfaction score for analgesic effect (SAE).

### Study hypothesis

(1) The experimental group will show greater VAS-P reduction and better control of intra-abdominal pressure (IAP) compared to the control group, indicating that interrupted epidural injections offer more effective pain relief and visceral protection.
(2) The incidence of adverse events in the experimental group will not be higher than in the control group, and the ICU-LOS and the 28D-MR will be shorter, indicating that the intermittent injection method is safe and may enhance patient outcomes.
(3) Analgesic satisfaction scores will be higher in the experimental group than in the control group, indicating that the nurse-led TEA management program better meets patient needs.

### Research team and implementation process

**Fig 3:**
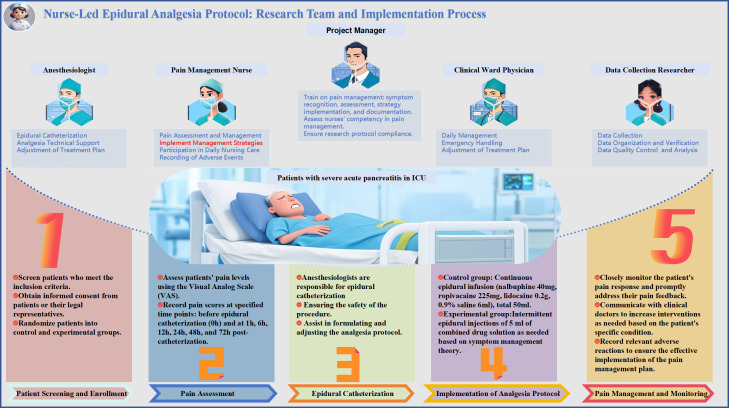
Nurse-Led Epidural Analgesia Protocol: Research Team and Implementation Process.

**Fig 4:**
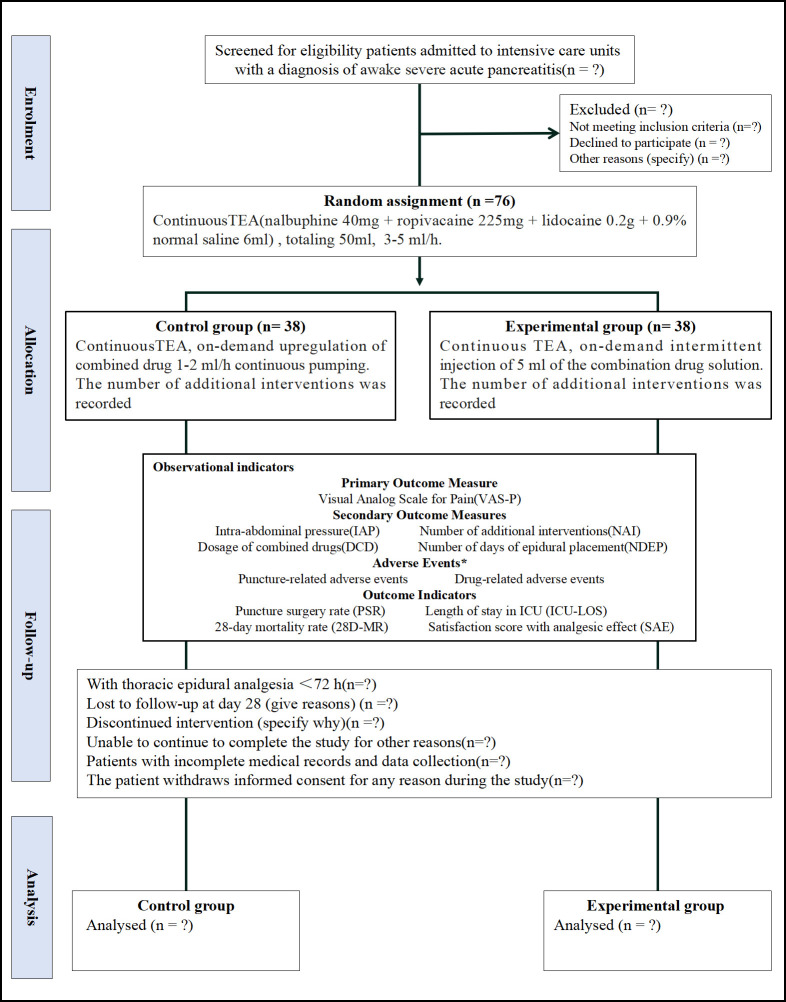
The patient study participation pathway.

The study team includes 14 multidisciplinary members, such as two project managers, two anesthesiologists, three ward clinical physicians, five pain management nurses, and two data collection researchers. The project managers handle training and supervising the implementation of the pain management strategy to ensure nurses are competent in applying it and meet the study protocol’s requirements. The anesthesiologists oversee epidural placement, provide technical support to ensure patient comfort and safety, and assist in developing the analgesic protocol. The ward clinical physicians manage daily patient care, handle emergencies, ensure safety, and participate in developing and adjusting the pain management program. Pain management nurses, based on SMT, lead pain assessment and management for SAP patients in the ICU, record pain scores, administer TEA drugs, respond to pain feedback, monitor pain responses closely, communicate promptly with physicians, ensure effective program implementation, and document adverse reactions. The data collection researchers gather study data, including pain scores and adverse events, ensure data accuracy and completeness, perform quality control and analysis using professional statistical software, and provide reports to uphold the scientific rigor of the study’s results. The implementation process includes: 1) screening and enrolling patients meeting criteria, obtaining informed consent, and randomizing groups; 2) assessing pain with the VAS and recording scores; 3) anesthesiologists perform epidural placement, ensuring safety; 4) administering the analgesic protocol with the control group receiving continuous epidural infusion, and the experimental group receiving intermittent epidural injections of local anesthetics as needed; 5) monitoring pain responses, adjusting treatment plans, analyzing data with statistical software to ensure reliable results, and preparing a comprehensive report. The nurse-led epidural analgesia program, along with the research team and implementation process, is shown in Fig 3. The patient study participation pathway is displayed in Fig 4.

### Study environment and population

This study protocol will be conducted at the Pancreatitis Diagnostic and Treatment Center in Guizhou Province, China. This multidisciplinary center works with various departments, including Anesthesia, Nursing, Traditional Chinese Medicine, Imaging, Ultrasound, Interventional Radiology, Nutrition, and Clinical Pharmacy. With two full-service ICUs and up to 70 beds, the center provides comprehensive care for pancreatitis patients, from consultation and examination to diagnosis, treatment, rehabilitation, and follow-up. It aims to offer patients convenient and accurate diagnostic and treatment services throughout the entire process. The study will include awake patients with SAP.

### Recruitment, screening, and enrollment

The research team will recruit SAP patients who meet the initial inclusion criteria in the ICUs. Recruitment information will be posted online, and the study will be introduced to patients or their legal representatives with the consent of the patients’ families to obtain their willingness to participate. For interested patients, members of the study team will conduct a detailed assessment based on the inclusion and exclusion criteria, including age, diagnosis, medical history, and laboratory results, to determine whether they qualify and to record reasons for exclusion. Patients who meet the criteria will be fully informed by the research team about the study’s purpose, methods, potential risks, and benefits. After confirming they understand and signing the informed consent form, they will be officially enrolled and randomly assigned to the control or experimental group. Throughout the process, the research team will strictly follow ethical guidelines, protect patients’ rights, and carefully document each step for subsequent data analysis and quality control.

### Inclusion criteria

(1) Volunteer to participate in the study and ensure the patient or their legal representative signs the informed consent SAP.
(2) A pain score (VAS-P) greater than 3, and patients are deemed to require TEA analgesia by the attending physician and anesthesiologist.
(3) Volunteer to participate in the study, with informed consent signed by the patient or their legal representative.e.

### Exclusion criteria

(1) Spinal deformity, contraindicating epidural puncture, or failure of thoracic epidural insertion.
(2) Patients with chronic SAP, patients with SAP combined with pregnancy, patients with pancreatic pseudocyst and infection.
(3) Patients with contraindications to epidural analgesia, such as coagulation disorders, local infection, allergy to lidocaine, etc.
(4) Patients with severe cardiac, pulmonary, hepatic, and renal failure; severe infection; malignant diseases; and autoimmune disorders.
(5) Patients with incomplete medical records.

### Dropout criteria

(1) The duration of epidural placement is less than 3 days.
(2) The patient withdraws informed consent at any time during the study for any reason.
(3) Unable to continue or complete the study due to reasons like a change in condition requiring transfer to or discharge from the hospital, refusal of further treatment, or evaluation.

### Sample size

This study protocol is a randomized controlled trial with the primary outcome measure being the VAS-P score. According to Sadowski’s 2015 study [9], continuous EA intervention showed significant improvement in subjective pain measured by the VAS (0–10 scale): pre-intervention 6.55 ± 3.39, after epidural administration 1.6 ± 1.838; Day 1: 0.57 ± 1.510; Day 2: 1.63 ± 3.46; Day 5: 1.86 ± 3.485; Day 7: 3 ± 2.380. These findings indicate that, in similar control protocols, the mean post-intervention VAS-P scores ranged from 0.57 to 3, with standard deviations from 1.510 to 3.485. Since no previous study has used the same intervention protocol, we hypothesize that the experimental group, which receives nurse-led intermittent withdrawal of epidural analgesia, will experience a reduction in average pain score of δ = 2 compared to the control group, representing the expected between-group difference. Based on the reported variability, we assume a pooled standard deviation of σ = 2.5 [3,11]. Using a two-sided significance level of α = 0.05 and a power of 90%, the sample size calculation, as per Formula 1, yields N = 33 participants per group. To account for a potential 15% dropout rate, a total of 76 participants will be needed, randomized in a 1:1 ratio.

***Formula 1:***


$$n=\frac{\text{2}(z_{\alpha }+z_{\beta }{)}^{\text{2}}\sigma^{\text{2}}}{\delta^{\text{2}}}$$

### Randomization

Participants in the study will be randomly assigned to either the control group or the experimental group using SPSS 29.0 software. An unblinded study coordinator, who will not perform any interventions or outcome assessments, will handle the randomization. Research staff, who are not involved clinically in the trial, will manage the random allocation list and prepare sealed, opaque, sequentially numbered envelopes, each containing a random number indicating the assigned treatment. Participants who meet the inclusion criteria will randomly select a sealed envelope containing the intervention protocol.

### Blinding

Since this is an interventional study, pain management nurses will not be informed of the specific patient groupings until after they start implementing the intervention. However, they will not be involved in other parts of the study. While efforts will be made to keep patients blinded to subgroups, complete concealment may not be possible. Therefore, data collectors and analysts will stay blinded to the specific intervention protocols. Independent personnel, who are unaware of the treatment assignments, will handle data collection and analysis to ensure that data collectors and statistical analysts remain blinded, reducing potential biases in data processing and analysis.

### Trial equipment

The trial equipment will include a micro syringe pump, medications (nalbuphine 20 mg/2 mL, ropivacaine 75 mg/10 mL, lidocaine 0.1 g/5 mL), and an epidural puncture kit. The micro syringe pump will accurately control the infusion rate and medication dosage, ensuring consistent and stable pain relief. It can also deliver the exact prescribed dose in a single administration. Nalbuphine acts as a potent analgesic by activating the κ opioid receptor. Ropivacaine, an intermediate local anesthetic of the amide class, provides local anesthesia and pain relief. Lidocaine, an amino amide-type local anesthetic, offers analgesic and anti-inflammatory effects. The epidural puncture kit includes a puncture needle, catheter, and other essential components to safely insert the catheter into the epidural space and ensure precise drug delivery.

### Baseline data collection

After patients are admitted to the ICU, eligible study subjects will be screened based on the inclusion and exclusion criteria. Before enrollment, baseline data will be collected, including patients’ general information such as age, gender, and time of admission. Clinical and diagnostic data will also be obtained, such as the etiology of onset, time from onset to admission, time from onset to ICU admission, time from onset to TEA, history of chronic diseases, related complications, type of SAP pathology, CTSI score, Ranson score, APACHE-II score, and major interventions during hospitalization. Additionally, pain scores and intra-abdominal pressure before epidural placement will be recorded. These baseline data will serve as comprehensive reference points for the subsequent evaluation of pain management outcomes.

### Study interventions

**Control group:** Continuous TEA will be administered with a drug mixture of nalbuphine 40 mg, ropivacaine 225 mg, lidocaine 0.2 g, and 0.9% saline 6 ml in a total volume of 50 ml at an infusion rate of 3–5 ml/h. If the patient’s VAS-P score exceeds 3 or the pain becomes intolerable, the infusion rate will be dynamically increased by 1–2 ml/h for continuous infusion. The number of additional interventions will also be recorded.

**Experimental group:** Based on continuous TEA, the pain management nurse will lead the TEA management strategy, incorporating STM. When the patient’s VAS-P exceeds a score of 3 or pain becomes intolerable, an epidural injection of 5 ml of the combined drug solution will be administered immediately, and the number of additional interventions will be recorded. The duration of each interval will be longer than 2 hours, and the patient’s pain response and feedback will be continuously monitored, allowing analgesic measures to be adjusted promptly.

#### Implementing personnel.

Trained pain management nurses will serve as program implementers, with the flexibility to customize analgesic measures for each patient’s specific needs. This strategy aims to control pain effectively while reducing unnecessary medication use. Close monitoring and prompt communication, led by the nurse, will ensure the program is well-executed and adjusted according to patient feedback and physician guidance, thereby enhancing patient comfort and safety.

### Observational indicators and measurements

Trained pain management nurses will serve as program implementers, with the flexibility to customize analgesic measures to each patient’s specific needs. This approach aims to manage pain effectively while minimizing unnecessary medication use. Close monitoring and prompt communication, led by the nurse, will ensure the program is appropriately executed and adjusted based on patient feedback and physician guidance, thereby enhancing patient comfort and safety.

**Visual Assessment Scale-Pain (VAS-P):** This uses a 100-mm straight line, with one end labeled “no pain at all” and the other end labeled “the most severe pain imaginable,” where the patient marks the position that best represents their pain level. Data collectors will record the data at specific time points (0 h, 1 h, 6 h, 12 h, 24 h, 48 h, 72 h). The 0 h measurement will be taken before epidural placement, and the remaining times will be after epidural placement at 1 h, 6 h, 12 h, 24 h, 48 h, and 72 h.

#### Secondary outcomes.

**Intra-abdominal pressure (IAP):** Measured indirectly through bladder pressure [31]. The procedure is as follows: The patient lies in a supine position. After inserting the indwelling catheter, connect it to a three-way valve and a pressure-measuring tube. Once the urine is drained, clamp the catheter and inject 25 mL of sterile saline at a temperature of 25°C to 35°C into the bladder. Align the measuring tape with the iliac crest and axillary midline to set the zero point. When the liquid level in the pressure measurement tube stops dropping and at the end of the patient’s exhalation, note the scale number at the concave surface of the fluid level; this indicates the intra-abdominal pressure. The timing and personnel for measurement are the same as those for VAS-P.

**Number of additional interventions (NAI):** This refers to the count of further interventions carried out by the pain management bedside nurses for patients with VAS-P greater than 3 or who experience intolerable pain after continuous TEA in both the control and experimental groups. The total interventions will be recorded on Days 1, 2, and 3, respectively.

**Dosage of combined drugs (DCD):** This refers to the total amount of combined drugs (nalbuphine 40 mg + ropivacaine 225 mg + lidocaine 0.2 g + 0.9% saline 6 ml, totaling 50 ml) used during epidural placement.

**Number of days of epidural placement (NDEP):** This will be calculated by the data collection researcher from the start of insertion to the time of extubation.

#### Adverse events (AE).

**Puncture-related AE:** Healthcare workers will observe and record the following eight indicators during daily care and examinations: spinal cord and nerve root injury, epidural hematoma, puncture site infection, total spinal anesthesia, headache after dural puncture, intradural infection, catheter blockage, catheter breakage, and ectasia. The number of AE cases will be counted during the period of epidural placement.

**Drug-related AE:** Pain management nurses will observe and record the following eight indicators after the patient’s medication: anaphylaxis, hypotension, nausea/vomiting, itching, tremor, sensory disturbances in the lower limbs, fever, decreased oxygen saturation, bradycardia, or tachycardia. The statistical methods and timings will be the same as those for puncture-related AE.

#### Outcome indicators.

**Puncture Success Rate (PSR):** This is calculated by dividing the number of successful punctures by the total number of punctures, as recorded by the data collector.

**Length of Stay in ICU (ICU-LOS):** The total number of days patients spend in the ICU will be recorded when they are transferred out.

**28-Day Mortality Rate (28D-MR):** This rate counts the number of patients who die within 28 days and calculates the corresponding mortality rate.

**Fig 5:**
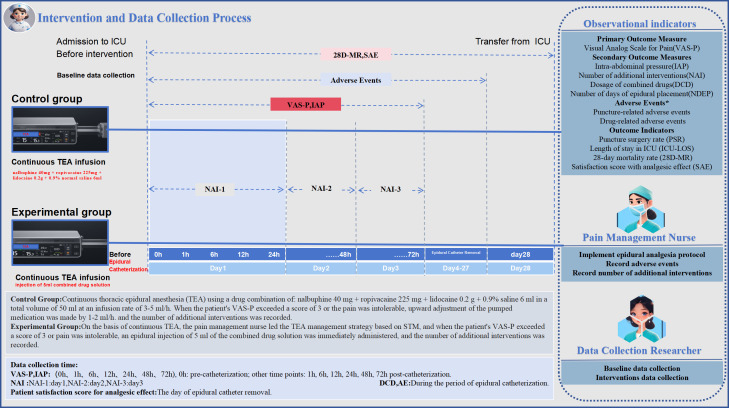
Intervention and Data Collection Process.

**Satisfaction with Analgesic Effect (SAE):** Patients’ satisfaction with the analgesic effect will be measured using a 0–10 scale, where zero indicates “very dissatisfied” and 10 indicates “very satisfied.” The data collector will record this information on the day the epidural tube is removed. Continuous variables include VAS-P, IAP, DCD, NDEP, NAI, and SAE; binary variables include puncture-related AE, drug-related AE, PSR, and 28D-MR. The intervention and data collection process is shown in Fig 5.

### Quality control

(1) All staff will complete standardized training and pass assessments to ensure consistent implementation of the intervention, reducing both executor and assessor bias.
(2) Collected data will be verified by two researchers before being entered into the system to ensure data accuracy.

### Data analysis

Data in this study will be analyzed using SPSS software (version 29, IBM Corp., Armonk, NY, USA). Baseline characteristics, including age, gender, admission time, and disease-related information, will be summarized with descriptive statistics: frequencies and percentages for categorical variables. The normality of continuous variables will be assessed using the Shapiro-Wilk test, histograms, and Q-Q plots. For normally distributed continuous variables, the mean ± standard deviation (SD) will be reported; for non-normally distributed data, the median and interquartile range (IQR) will be used.

Between-group comparisons will be conducted using t-tests, Mann-Whitney U tests, or chi-square/Fisher’s exact tests, depending on the variable type and distribution. Repeated measures data, such as VAS-P scores at 0h, 1h, 6h, 12h, 24h, 48h, and 72h, will be analyzed with a multivariate mixed-effects repeated measures model to handle data missing at random. Secondary outcomes, including IAP and NAI, will be analyzed similarly. Other continuous outcomes, such as DCD, NDEP, SAE, and ICU-LOS, will be summarized as mean ± standard deviation or median with interquartile range and compared between groups accordingly.

Missing data will first be sought through a retrospective review of medical records. If unavailable, data missing at random will be handled with multiple imputation, while data not at random will be analyzed using a pattern mixture model. The choice of method will consider data distribution and the mechanism of missingness to ensure reliability. Statistical significance will be set at α = 0.05.

### Data management and monitoring

Data management and monitoring will follow both national and international good clinical practice standards, along with all relevant regulatory and ethical guidelines. All adverse events, whether related or unrelated to the study, will be systematically documented in source records. Throughout the study, ongoing assessments will be conducted to maintain research quality and ensure regulatory compliance. To preserve independence and objectivity, monitoring personnel will not participate in the direct execution of the study. The Ethics Committee will oversee the monitoring process, verify investigators’ and study staff’s qualifications, and confirm the accuracy and appropriateness of all documents. Data quality will be meticulously checked using various methods to ensure its accuracy, consistency, and completeness.

### Ethics and dissemination

The study protocol (version 1.0, dated January 1, 2025) has been approved by the Ethics Committee of Zunyi Medical University Hospital. The study will adhere to both national and international ethical standards to ensure regulatory compliance. All serious adverse events will be carefully documented, regardless of their relation to the study. Ongoing assessments will monitor research quality and regulatory adherence. Independent monitoring personnel will not participate in the study’s conduct to maintain objectivity. The Ethics Committee will also approve the monitoring procedures, including verifying investigator qualifications and ensuring proper documentation. Study results will be shared with the global scientific community through peer-reviewed journals.

## Discussion

### Pain mechanisms and challenges in managing SAP

According to the 2012 Atlanta Consensus on Pancreatitis, acute pancreatitis is classified into mild (MAP), moderately severe (MSAP), and severe (SAP) [32,33]. SAP progresses rapidly, often accompanied by peripancreatic tissue necrosis and/or organ failure, and is very difficult to treat, with morbidity and mortality rates of up to 30% [34]. The pain mechanism in patients with SAP is highly complex, mainly involving neurogenic inflammation and harmful peripheral tissue stimuli, with pain showing significant fluctuation and irregularity, which greatly increases the difficulty of pain management [1]. Visceral pain is usually triggered by tissue inflammation, ischemia, or distension, which not only causes organ damage but is also closely linked to emotional distress and a decline in the patient’s quality of life [35]. Multiple factors, including increased pancreatic duct pressure, duct obstruction, and the buildup of inflammatory cells around nerves, influence the fluctuation of pain [36]. Neurogenic inflammation activates primary afferent neurons, releasing neuropeptides such as CGRP and substance P, which enhance spinal excitability and amplify pain signals [37]. Additionally, the vicious cycle between inflammation and pain is repeatedly triggered under different conditions and times, resulting in ongoing fluctuations in pain. This not only worsens the patient’s suffering but also complicates pain management. Overall, pain in SAP patients arises not only from physical damage but is also affected by neurobiological and psychological factors, making it more challenging to develop effective clinical pain management strategies [38].

### Application and potential value of nurse-led TEA strategy in SAP pain management

TEA is a minimally invasive technique that involves administering low-dose local anesthetics through an epidural catheter, allowing infiltration into the connective tissue around the nerve roots [39]. This results in a reversible blockade of regional nerve conduction and sympathetic activity. In the context of SAP, TEA has demonstrated potential benefits for early management [8,14,15,34,40]. Evidence from both animal studies and clinical research supports its effectiveness in reducing pain, and its use has been recommended in the 2022 edition of the Chinese expert consensus. However, while continuous TEA infusion can reduce pain, some patients may still require additional intermittent local anesthetic injections, indicating that a single continuous TEA regimen might not meet every patient’s individual needs. On-demand intermittent injections can serve as a practical addition, offering a more flexible and personalized approach to managing SAP pain.

In the ICU setting, nurses play a crucial role in SAP management [41]. Their ongoing monitoring, specialized skills, and quick response capabilities allow for dynamic adjustments of analgesic interventions, addressing the limitations of traditional TEA management in terms of individualization [42,43]. A nurse-led TEA approach based on SMT, utilizing standardized and personalized intermittent injection protocols, has the potential to improve pain control, stabilize fluctuations in intra-abdominal pressure, reduce additional interventions, and enhance patient satisfaction. This study aims to assess the safety and effectiveness of this method, providing essential evidence for adopting individualized pain management strategies and highlighting the innovative role of nurses in SAP pain control.

#### Innovation.

This protocol is the first effort to incorporate SMT into nurse-led TEA for SAP. By providing a structured approach to symptom assessment and intervention, SMT may improve pain management and support personalized care. The proposed strategy, which combines continuous TEA with on-demand intermittent injections, likely offers greater flexibility and effectiveness than traditional methods. These innovations could not only enhance pain control in SAP but also reinforce the vital role of nurses in pain management and promote wider use of SMT in critical care settings.

### Limitations

This study protocol will be carried out as a single-center trial, which may limit how broadly the results can be applied in the future. Although the planned sample size calculation is intended to ensure statistical validity, it might still be too small to detect minor but clinically important differences. Pain will be assessed using the VAS-P, but this measure could be affected by patients’ subjective interpretation. There is also a risk of missing data due to patient withdrawal or other unforeseen issues, which could impact the strength of the final results. Additionally, the study duration may not be sufficient to fully observe the long-term effects of the intervention.

### Practical significance

Despite its limitations, this study protocol provides significant practical value. Exploring the potential benefits of SMT-based nurse-led pain management paves the way for a more personalized and effective approach to managing pain for patients with SAP. If the experimental group shows better outcomes, this could prompt a shift in clinical practice toward using intermittent epidural injections instead of continuous TEA. Such a change would not only improve patient comfort and satisfaction but also potentially lower healthcare costs linked to longer ICU stays and adverse events.

### Patient and public involvement

Patients and/or the public were not involved in the design, conduct, reporting, or dissemination plans of the current research.

## Supporting information

S1 File Research Protocol. (PDF)

S2 File S1 SPIRIT-Checklist. (PDF)
