# Supplementary material for: Effectiveness and safety of nurse-led different epidural analgesia methods based on symptom management theory in patients with severe acute pancreatitis: A single-center RCT protocol
Source: PLoS One. 2026 Jan 2;21(1):e0337803. doi: 10.1371/journal.pone.0337803 (PMC12758678; doi:10.1371/journal.pone.0337803)
Supplement: S1 File — (PDF) [file pone.0337803.s001.pdf]

Effectiveness and Safety of Different Epidural Administration Methods Led by Nurses Based on Symptom Management Theory for Pain Management in Severe Acute Pancreatitis: A Clinical Study

Research Protocol

Contents

I. Research Background and Current Status 2
II. Research Introduction 4
1. Research Title 4
2. Initiating Institution of the Study 4
3. Study Duration 4
4. Research Center 4
5. Research Purpose 4
6. Research Content 5
7.Study Hypothesis 5
8.Theory evidence 6
9.Research team and implementation process 7
10.Study environment and population 9
11.Recruitment, screening, and enrollment 10
12.Sample size 11
13.Randomization 11
14.Blinding 11
15.Trial Equipment 12
16.Data collection process 12
17.Study interventions 12
18.Implementing personnel 13
19.Primary outcome measure 13
20.Secondary outcomes 13
21.Adverse Events 13
22.Outcome Indicators 14
23.Quality control 14
24.Data analysis 15
25.Data management and monitoring 16
26. Innovation: 16
27. Limitations: 16
28. Practical Significance: 17
29. Risks and Safety Measures of This Study 17
30. Rights, Benefits, and Compensation of Subjects Participating in This Study 18
32. Privacy and Confidentiality of Subjects 19
33. Publication or Disclosure of This Study's Results 20
34. Implementation Conditions and Researchers of This Study 20
35. SPIRIT-Figure 21

I. Research Background and Current Status

Acute pancreatitis (AP) is one of the common acute abdominal conditions in clinical practice, which may be associated with the failure of one or multiple organ functions. In high-income countries, the annual incidence rate is 34 cases per 100,000 person-years. The incidence of AP is on the rise worldwide, imposing a significant burden on healthcare and society. According to the revised Atlanta Classification of Pancreatitis in 2012, AP is categorized into three grades: mild acute pancreatitis (MAP), moderate severe acute pancreatitis (MSAP), and severe acute pancreatitis (SAP). These three types of pancreatitis have different clinical courses and outcomes. Most patients with MAP recover within one week, while those with SAP and MSAP have a more rapid disease progression, often accompanied by necrosis of peripancreatic tissues or (and) organ failure. The treatment is challenging, and the mortality rate is as high as 30%. Despite improvements in treatment and intensive care, SAP remains associated with high mortality. Therefore, early de-escalation of severity in SAP is crucial for reducing complications and improving prognosis.

The etiology of AP, such as secondary to gallstones (the most common cause), alcohol, endoscopic retrograde cholangiopancreatography (ERCP), and various drugs, leads to pathological cell pathways and organelle dysfunction, ultimately causing acinar cell death and triggering local and systemic inflammatory responses. The pathogenesis of AP has not been fully elucidated, and currently, the trypsinogen theory is dominant in the development of SAP. This theory posits that various etiologies lead to the activation of trypsinogen, which in turn activates a series of digestive enzymes, resulting in autodigestion and damage to the pancreas. A large number of proteolytic enzymes trigger local inflammation in the gland, and the pancreas continuously releases pro-inflammatory cytokines such as interleukin IL-1, IL-6, and IL-8, and TNF-α, inducing a systemic inflammatory state. This subsequently leads to strong activation of the sympathetic nervous system, causing a reduction in blood flow to the gastrointestinal system, which in turn leads to pancreatic ischemic necrosis, systemic inflammatory response syndrome (SIRS), multiple organ dysfunction syndrome (MODS), and acute respiratory distress syndrome (ARDS), pleural effusion, hypovolemia, renal failure, and even death. Although the mortality rate of acute pancreatitis has decreased in recent years due to improved diagnostic and nursing levels, the incidence and long-term sequelae remain a significant challenge for clinicians.

Persistent upper abdominal pain and pain radiating to the back are the earliest and most common symptoms of AP. Adequate pain management is crucial for SAP management. The pain in SAP patients can progress from an initial 1-3 days of persistent vague pain to severe abdominal pain lasting for several days or weeks. If pain is not

controlled in a timely and effective manner, it will affect the patient's comfort and recovery progress, and may even impact the patient's mood. The use of intravenous opioids for pain relief is associated with corresponding persistent side effects, such as respiratory depression, somnolence, delirium, myoclonus, nausea, vomiting, constipation, immune suppression, endocrine disorders, and addiction, among many other adverse reactions.

Thoracic epidural anesthesia (TEA) involves intermittent injection of low-concentration local anesthetics through a catheter into the epidural space, surrounding the connective tissue around the nerve roots, and blocking the regional nerve roots through local infiltration. It is a minimally invasive treatment method that reversibly controls the activity of sympathetic nerves within the region. TEA is a widely used anesthetic technique that can produce extensive visceral sympathetic and sensory nerve block effects, with roles in pain relief, anti-inflammation, and vasodilation to improve visceral organ perfusion. In addition to providing good anesthesia and analgesia, TEA also has many non-analgesic effects. Studies have shown that intraoperative and postoperative local application of anesthetics in the epidural space can prevent a reduction in visceral microvascular perfusion and shorten the recovery time of gastrointestinal function. In animal experiments conducted by Wang X et al., it was found that thoracic epidural anesthesia significantly promotes distal gastric microcirculatory perfusion and enhances the vitality of the stomach and intestines. Wang Huiying's research indicated that TEA can reduce the impact on gastrointestinal perfusion during endotoxemia while ensuring hemodynamic stability. It was also found that thoracic epidural anesthesia promotes gastric mucosal blood flow during sepsis. Paralytic ileus is a common complication of acute pancreatitis, and thoracic epidural anesthesia can improve paralytic ileus by blocking nociceptive afferent nerves and thoracolumbar sympathetic efferent fibers to maintain the function of parasympathetic efferent fibers. In addition to its analgesic effects, the regulatory role of TEA can also improve organ perfusion, delay intestinal acidosis in hypoxia, increase intramucosal pH in patients with peritonitis, thereby reducing postoperative complications, shortening hospital stay, and improving survival rates.

TEA is recommended as one of the early analgesic methods for acute pancreatitis due to its good analgesic effect and improvement of the prognosis of patients with acute pancreatitis. Although studies have shown benefits of TEA in acute pancreatitis in animals and humans, it has been clinically observed that some patients still experience unrelieved pain after continuous epidural infusion of analgesics (combination solution: 40 mg of nalbuphine, 225 mg of ropivacaine, 0.2 g of lidocaine, and 6 ml of 0.9% normal saline, totaling 50 ml), and require additional on-demand epidural combination solution (5 ml) to achieve effective pain relief. There is currently no definitive epidural analgesic regimen, lacking specificity and individualization, making it difficult to meet the unique needs of different patients. In the ICU setting, nurses, as the primary managers of patient pain, play a crucial role in pain

assessment, drug administration, and complication monitoring. However, the management plan for epidural drug administration in SAP patients still needs further optimization.

Therefore, this study, based on the Symptom Management Theory (SMT) and led by nurses, aims to objectively evaluate the effectiveness and safety of continuous epidural infusion compared with intermittent epidural combination solution (5 ml) on demand. The goal is to provide an optimized pain management plan for SAP patients. The research findings may offer potential clinical evidence for pancreatitis analgesic treatment and have significant implications for improving the prognosis of SAP.

This study is the first to explore the application of Symptom Management Theory (SMT) in pain management for patients with severe acute pancreatitis (SAP) led by nurses. Integrating SMT into pain management provides a structured approach for symptom assessment and intervention, which may enhance pain control. The study also introduces a flexible analgesic regimen, combining continuous thoracic epidural analgesia (TEA) with intermittent injections on demand, aiming to offer more personalized and effective pain management. These innovations may provide new insights for optimizing pain management plans for SAP patients and promote the role of nurses in pain management as well as the application of SMT in other clinical settings.

II. Research Introduction

1. Research Title

Effectiveness and Safety of Different Epidural Administration Methods Led by Nurses Based on Symptom Management Theory for Pain Management in Severe Acute Pancreatitis: A Clinical Study

2. Initiating Institution of the Study

Affiliated Hospital of Zunyi Medical University. The leading unit of the study is the Affiliated Hospital of Zunyi Medical University. The department undertaking the study is the Department of Critical Care Medicine, and the principal investigator is Yuan You.

3. Study Duration

January 2025 to February 2026.

4. Research Center

Department of Critical Care Medicine, Affiliated Hospital of Zunyi Medical University.

5. Research Purpose

Given that animal experiments and clinical studies have shown additional benefits of thoracic epidural anesthesia

(TEA) for acute pancreatitis (AP), the efficacy of TEA in treating AP has garnered significant attention in recent years. The current expert consensus on analgesic treatment for severe acute pancreatitis (SAP) in China (2022 edition) also recommends the use of TEA for pain relief. However, clinical observations have revealed that some patients still experience unrelieved pain after continuous epidural infusion for analgesia and require additional on-demand drug injections to achieve significant pain reduction. Currently, there is no unified and clear protocol for epidural analgesia administration, which generally lacks specificity and individualization, making it difficult to fully meet the personalized needs of different patients. Particularly in the intensive care unit (ICU) setting, nurses, as the primary managers of patient pain, play a crucial role in pain assessment, drug administration, and complication monitoring. Therefore, there is an urgent need to develop and optimize more targeted and individualized management plans for epidural drug administration in SAP patients. This study aims to explore and compare the efficacy and safety of different epidural drug administration methods in pain management to provide an optimized pain management strategy for SAP patients.

6. Research Content

This study was a single-center, randomized, controlled clinical trial protocol planned to include 76 patients with SAP who were randomly divided into two groups in order to assess the efficacy and safety of different SMT-based nurse-led epidural analgesia delivery modalities. In the control group, continuous TEA was used with a combination of nalbuphine 40 mg, ropivacaine 225 mg, lidocaine 0.2 g, and 0.9% saline 6 ml in a total of 50 ml, which was pumped at a rapid duration of 3-5 ml/h. When the patient's VAS-P exceeded 3 points or when the patient was unable to tolerate pain, the pumping of the combined solution was appropriately adjusted upward by 1-2 ml/h. In the experimental group, on the basis of continuous TEA, a pain management nurse led the TEA pain management strategy according to the SMT, reported to the doctor when the VAS-P exceeded 3 points or when the patient was unable to tolerate pain, and injected 5 ml of the combined drug solution intermittently with an intermittent interval time of greater than 2 hours each time, as per the doctor's prescription. Hours. The patient's pain response and feedback were also continuously monitored for timely adjustment of analgesic measures. The primary outcome indicator was VAS-P, and secondary outcome indicators included intra-abdominal pressure(IAP), number of additional interventions(NAI), dosage of combined drugs(DCD)， number of days of epidural placement(NDEP), and 16 adverse events(AE). Outcome indicators included puncture success rate(PSR), length of stay in ICU (ICU-LOS), 28-day mortality rate (28D-MR), and patient satisfaction score for analgesic effect(SAE).

7.Study Hypothesis

(1) The experimental group will be superior to the control group in terms of VAS-P reduction and IAP control,

suggesting that intermittent epidural injections provide superior pain management and visceral protection.

(2) The incidence of adverse events in the experimental group will not be higher than that in the control group, and the length of hospitalization in the ICU and 28-day mortality will be lower, suggesting that the intermittent injection method is safe and may improve prognosis.

(3) Analgesic satisfaction scores will be higher in the experimental group than in the control group, suggesting that the nurse-led pain management program is more responsive to patient needs.

8.Theory evidence

Based on clinical practice, the team searches domestic and international databases to summarize the best evidence based on evidence-based, and develops pain management strategies based on the SMT theoretical framework. The theory focuses on managing patients' symptoms and consists of symptom experience, symptom management strategies and symptom outcomes. In the ICU, in response to pain in patients with SAP, SMT provides a structured approach to identifying pain symptoms by assessing pain levels on a regular basis using the VAS-P or patient self-report, recording scores, and thus understanding the patient's symptom experience. Based on these assessments, a 6W2H pain symptom management strategy was developed to individualize symptom management. The strategy consisted of two regimens of continuous epidural pumping of combination medications and on-demand intermittent epidural injections of 5 ml of combination medications on top of this. The key elements of why (purpose), who (in charge), who (subject), where (location), when (timing), what (measures), how (methods), and how much (frequency) were specified. Symptom outcome refers to the assessment of the effectiveness of the symptom management strategy after its implementation. In this study, the effectiveness of pain management was assessed primarily through indicators such as pain scores, dosage of combination medications, incidence of adverse events, length of stay in the ICU, mortality, and patient pain satisfaction. By recording and analyzing these indicators, the effectiveness and safety of different analgesic regimens were assessed to provide a scientific basis for optimizing pain management regimens. A comprehensive baseline assessment of other factors affecting pain, including person, environment and health/illness will be conducted to ensure consistent patient inclusion criteria. The SMT-based pain management framework for SAP patients is shown in *Figure 1.*

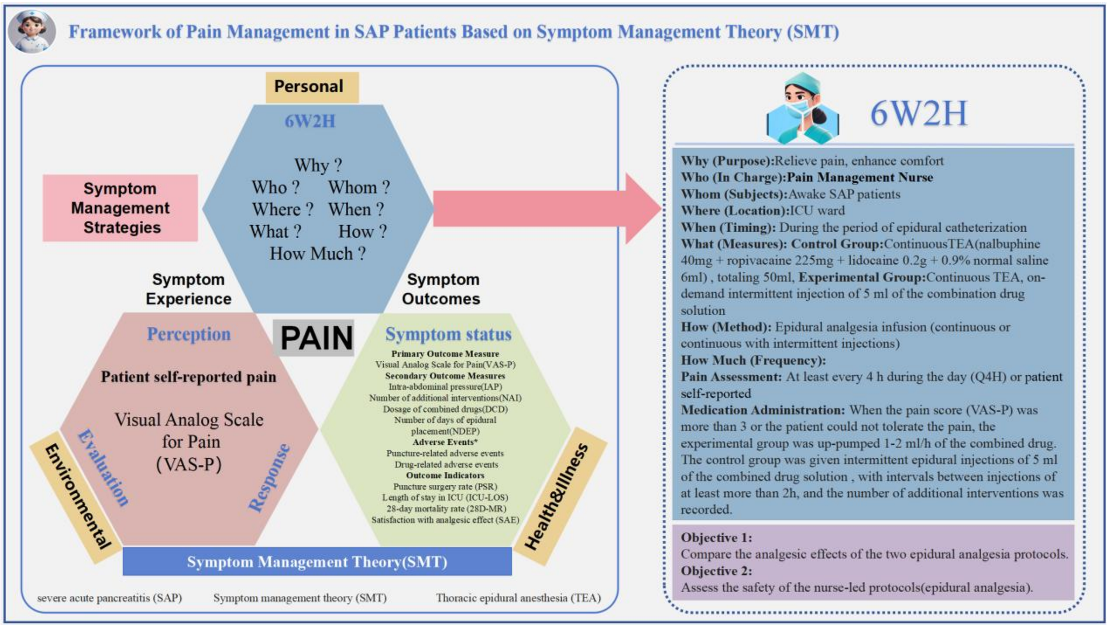

Figure 1:Framework of Pain Management in SAP Patients Based on Symptom Management Theory (SMT)

9.Research team and implementation process

The study team consisted of 14 multidisciplinary members, including two project managers, two anesthesiologists, three ward clinical physicians, five pain management nurses, and two data collection researchers.

The project manager was responsible for the training and supervision of the pain management strategy to ensure that the nurses were competent in implementing the strategy and meeting the requirements of the study protocol. The anesthesiologist was responsible for epidural placement, providing technical support to ensure patient comfort and safety, and assisting in the development of the analgesic protocol. The ward clinical physicians were responsible for the daily medical management of patients, handling emergencies, ensuring patient safety, and participating in the development and adjustment of the pain management program. Pain management nurses, based on SMT, lead the implementation of pain assessment and management strategies for SAP patients in the ICU, record pain scores, perform TEA drug administration, handle patients' pain feedback, closely observe pain responses, communicate with physicians in a timely manner, ensure effective implementation of the pain management program, and record adverse reactions. The data collection researcher is responsible for collecting study data, including pain scores and adverse events, ensuring data accuracy and completeness, performing data quality control and analysis, using professional statistical software to analyze the data, and providing data analysis reports to ensure the scientific and rigorous nature of the study results. The implementation process includes: 1. patient screening and enrollment: screen patients who meet the criteria, obtain informed consent, and randomize the group; 2. pain assessment: use VAS to assess pain and record scores; 3. epidural placement: anesthesiologists perform placement and ensure safety; 4. implementation of analgesic program: the control group uses continuous

epidural infusion, and the experimental group performs intermittent epidural injection of local anesthetics on demand; 5. pain management and monitoring: monitor the pain response, and provide data analysis using professional statistical software to ensure that the results of the study are scientific and provide a report. Pain management and monitoring: monitoring pain reactions, handling feedback, adjusting the treatment plan, and recording adverse reactions. Nurse-led epidural analgesia program: the research team and implementation process is shown in *Figure 3*.the consort diagram of the trial is shown in *Figure 4*.

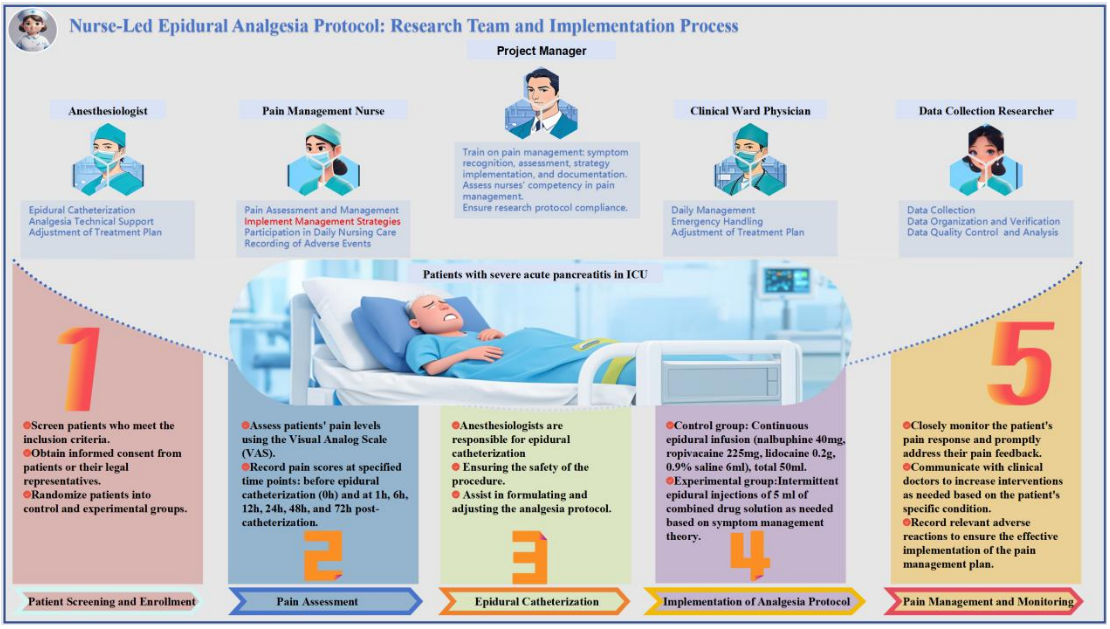

Figure 2: Nurse-Led Epidural Analgesia Protocol: Research Team and Implementation Process

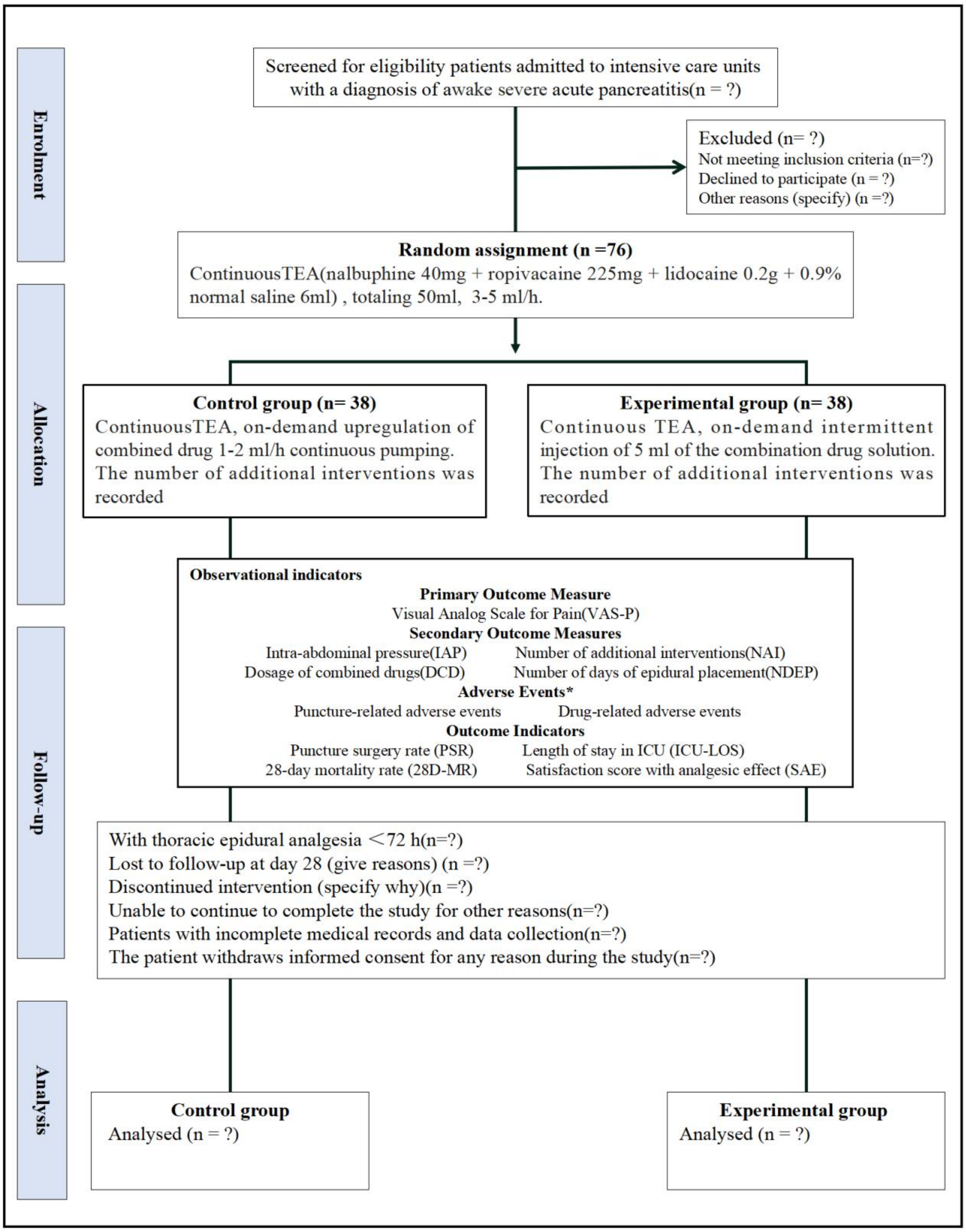

Figure3: The patient study participation pathway

10.Study environment and population

This study was conducted in the Pancreatitis Diagnostic and Treatment Center of Guizhou Province, China, which is a multidisciplinary center in collaboration with Anesthesia, Nursing, Traditional Chinese Medicine, Imaging, Ultrasound, Intervention, Nutrition, Clinical Pharmacy, etc. With two comprehensive ICUs and a total number of beds up to 70, this center is dedicated to comprehensively cover the whole process of pancreatitis patients from the

consultation, examination, diagnosis, treatment, rehabilitation, and follow up, and to provide the patients with convenient whole process of precise diagnostic and treatment services. The population of this study consisted of awake SAP patients.

11.Recruitment, screening, and enrollment

The research team will recruit SAP patients who meet the initial inclusion criteria in the ICU, and the recruitment information will be announced through the Internet, and the study will be introduced to the patients or their legal representatives with the consent of the patients' families to obtain the intention to participate. For interested patients, members of the study team will conduct a detailed assessment based on the inclusion and exclusion criteria, including age, diagnosis, medical history, and laboratory findings, to determine whether they meet the inclusion criteria and record the reasons for exclusion. Patients who meet the inclusion criteria will be introduced in detail by members of the research team with information on the purpose, methods, potential risks and benefits of the study, and after ensuring that they fully understand and sign the informed consent form, they will be formally enrolled in the study and entered into the control or experimental group according to the random allocation scheme. Throughout the process, the research team will strictly follow the ethical principles, protect patients' rights and interests, and record the operation process and results of each step in detail for subsequent data analysis and quality control.

Inclusion criteria

(1) Patients aged ≥18 years and ≤65 years.

(2) Patients with a clear diagnosis of SAP.

(3) Pain score (VAS-P) greater than 3.

(4) Informed consent signed by the patient or his/her legal representative.

Exclusion criteria

(1) Spinal deformity, inappropriate for epidural puncture, failure of thoracic epidural puncture.

(2) Patients with chronic SAP, patients with SAP combined with pregnancy, patients with pancreatic pseudocyst and infection.

(3) Patients with contraindications to epidural analgesia, such as coagulation disorders, local infection, allergy to lidocaine, etc.

(4) Combined with severe cardiac, pulmonary, hepatic and renal failure, serious infection, malignant diseases and autoimmune diseases.

(5) Patients with incomplete medical records.

Dropout criteria

(1)The duration of epidural placement is less than 3 days.

(2) The patient withdraws informed consent for any reason during the study.

(3)Unable to continue to complete the study for other reasons, such as a change in condition requiring transfer to or discharge from the hospital or refusal of further treatment or evaluation.

12.Sample size

This study is a randomized controlled trial, and the primary outcome indicator is the VAS-P score. From the literature review, it is known that the mean VAS-P scores after the intervention of similar control group protocols are 1-3, and there is no study similar to the experimental group, and the mean VAS-P scores of the pre-experimental group are 1-2, It is proposed to formulate the standard deviation of the two groups combined $\sigma = 2.5$, and it is expected that the pain symptom scores of the experimental group can be decreased by $\delta = 2$. Setting a bilaterality of $\alpha = 0.05$, the degree of certainty is 90%, and based on the formula of the sample size *Formula 1,* the sample size is calculated for each group of N = 33 participants. Assuming a 15% dropout rate, a total of 76 participants were needed based on a 1:1 randomization ratio.

Formula 1:

$$n = \frac{2(z_\alpha + z_\beta)^2 + \sigma^2}{\delta^2}$$

13.Randomization

The study participants were evenly divided into the control group and experimental group using random numbers generated with SPSS 29.0 software. Randomization will be performed by an unblinded study coordinator who does not perform any intervention or outcome assessments. Research staff, with no clinical involvement in the trial, will be responsible for keeping the random allocation list and preparing sealed, opaque sequentially numbered envelopes, each containing a random number denoting the allocated treatment. Participants meeting the inclusion criteria will randomly select a sealed envelope containing the intervention protocol.

14.Blinding

Because this is an interventional study, pain management nurses will not be aware of the specific grouping of patients until after they begin to implement the intervention, but they will not be involved in other parts of the study. Although there are measures that can be taken to try to keep patients blinded to subgroups, there are still difficulties in avoiding exposure altogether. For this reason, data collectors and data analysts will remain blinded to the specific intervention protocols, and independent persons who are not aware of the specific intervention protocols will be responsible for data collection and analysis, thus ensuring that data collectors and statistical

analysts are blinded to the treatment assignment and minimizing potential bias in data processing and analysis.

15.Trial Equipment

The test equipment included a micro syringe pump, drugs (nalbuphine 20mg/2ml, ropivacaine 75mg/10ml, lidocaine 0.1g/5ml) and an epidural puncture placement kit. The micro syringe pump is used to accurately control the pumping rate and dosage of medication to ensure that the medication is pumped consistently and uniformly to maintain a stable analgesic effect. It is also possible to regulate the exact dose to be injected at one time. Nalbuphine acts as a potent analgesic by agonizing the κ opioid receptor; Ropivacaine is an amide-type intermediate local anesthetic that provides local anesthesia and analgesia; Lidocaine is an amino-amide-type local anesthetic that provides analgesia and anti-inflammatory effects. The Epidural Puncture Placement Kit contains a puncture needle, catheter, etc., which is used to safely place the catheter into the epidural space to nsure accurate drug pumping.

16.Data collection process

After patients were admitted to the ICU, eligible study subjects were screened according to the inclusion and exclusion criteria. Before enrollment, the following baseline data were collected: patients' general information: including age, gender, and time of admission; important clinical and diagnostic information: including etiology of onset, time from onset to admission, time from onset to admission to the ICU, time from onset to TEA, history of previous chronic diseases, related complications, type of SAP pathology, CTSI score, Ranson score, APACHE-II score and major interventions during hospitalization; and pain score and intra-abdominal pressure before epidural placement. These baseline data cover the basic characteristics, clinical status and pain of the patients and provide a comprehensive reference for subsequent assessment of pain management outcomes.

17.Study interventions

Control group：Continuous TEA using a drug combination of: nalbuphine 40 mg + ropivacaine 225 mg + lidocaine 0.2 g + 0.9% saline 6 ml in a total volume of 50 ml at an infusion rate of 3-5 ml/h. When the patient's VAS-P exceeded a score of 3 or the pain was intolerable, upward adjustment of the pumped medication was made by 1-2 ml/h. and the number of additional interventions was recorded.

Experimental group： On the basis of continuous TEA, the pain management nurse led the TEA management strategy based on STM, and when the patient's VAS-P exceeded a score of 3 or pain was intolerable, an epidural injection of 5 ml of the combined drug solution was immediately administered, and the number of additional interventions was recorded. The duration of each interval was greater than 2 hours, and the patient's pain response and feedback were continuously monitored so that analgesic measures could be adjusted in a timely manner.

18.Implementing personnel

Trained pain management nurses act as program implementers, with the flexibility to adapt analgesic measures to the patient's specific situation. This approach aims to effectively control pain while reducing unnecessary medication use. Close monitoring and timely communication led by the nurse ensures that the pain management program is effectively implemented and adjusted based on patient feedback and physician guidance, thereby improving patient comfort and safety.

Observational indicators and measurements

19.Primary outcome measure

Visual Assessment Scale-Pain (VAS-P): which consists of a 100-mm straight line, with one end indicating “no pain at all” and the other end indicating “the most severe pain imaginable”, with the patient marking the corresponding position on the line to represent his/her pain level. Patients marked the corresponding position on the line to represent their pain level.The data were collected by the data collectors at the specified time points (0h, 1h, 6h, 12h, 24h, 48h, 72h). Among them, 0h was before epidural placement, and the other times were 1h, 6h, 12h, 24h, 48h and 72h after epidural placement.

20.Secondary outcomes

Intra-abdominal pressure (IAP):Measured by transcystic intra-abdominal pressure measurement method. Specific operation is: the patient lying position, after indwelling catheter connected to the tee and pressure measuring tube, after draining the urine clamped closed urinary catheter, through the urinary catheter into the bladder 25 ℃ ~ 35 ℃ 25 ml of sterile saline, measuring tape aligned with the iliac crest axillary midline as the zero adjustment, when the liquid plane in the pressure measuring tube is no longer falling, in the end of the patient's exhalation, the scale number of the concave surface of the liquid surface in the pressure measuring tube to the scale number of the pressure is the intra-abdominal pressure. Measurement time and personnel are the same as the VAS-P.

Number of additional interventions(NAI): the number of additional interventions given by the pain management bedside nurses to patients with VAS-P greater than 3 or intolerable pain after continuous TEA in the control group and experimental group.The total number of interventions was recorded on Day1, Day2, and Day3, respectively.

Dosage of combined drugs(DCD): the total amount of combined drugs (nalbuphine 40 mg + ropivacaine 225 mg + lidocaine 0.2 g + 0.9% saline 6 ml, totaling 50 ml) during epidural placement.

Number of days of epidural placement(NDEP): calculated by the data collection researcher from the start of insertion to the time of extubation.

21.Adverse Events

Puncture-related AE : Healthcare workers observe and record the following 8 indicators during daily care and examination: spinal cord and nerve root injury, epidural hematoma, puncture site infection, total spinal anesthesia, headache after dural puncture, intradural infection, catheter blockage, catheter breakage and ectasia. The number of cases of AE was counted for the period of epidural placement.

Drug-related AE: Pain management nurses observed and recorded the following 8 indicators after the patient's medication: anaphylaxis, hypotension, nausea/vomiting, itching, tremor, sensory disturbances in the lower limbs, fever, decreased oxygen saturation, bradycardia or tachycardia. The statistical methods and times were the same as those for puncture-related AE.

22.Outcome Indicators

Puncture surgery rate (PSR): Calculate the ratio of the number of successful puncture cases to the total number of punctures, which was counted by the data collector.

Length of stay in ICU (ICU-LOS): when patients are transferred out of ICU, the total number of days of ICU stay is recorded.

28-day mortality rate (28D-MR): count the number of patients who died within 28 days and calculate the 28-day mortality rate.

Satisfaction with analgesic effect (SAE): patients' satisfaction with the analgesic effect was assessed using a 0-10 scale, with 0 indicating very dissatisfied and 10 indicating very satisfied. It was collected by the data collector on the day of epidural tube removal.The intervention and data collection process are shown in *Figure 4.*

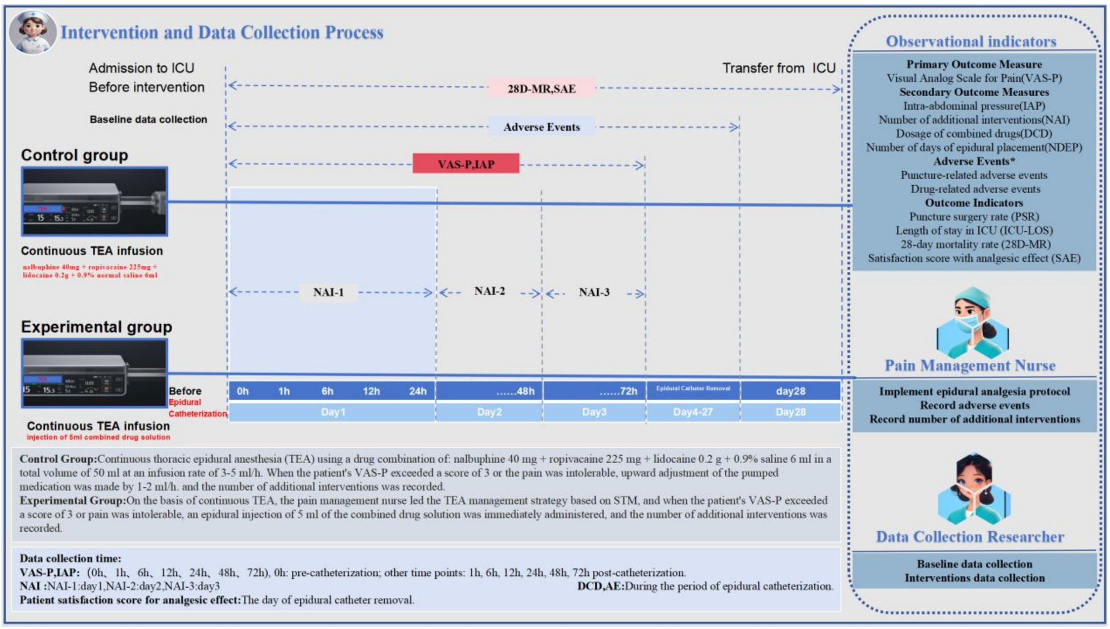

Figure 5:Intervention and Data Collection Process

23.Quality control

(1) All staff must undergo standardized training and pass assessments to ensure consistent implementation of the intervention, minimizing both executor and assessor bias.

(2) Collected data will be cross-verified by two researchers before being entered into the system to ensure data accuracy.

24.Data analysis

Data will be analyzed in this study using GraphPad Prism software to ensure accuracy and consistency of results. For baseline characteristics data, patients' age, gender, admission time, and disease-related clinical information will be descriptive statistics, Categorical variables will be presented as frequencies and proportions, whereas normally distributed continuous variables will be described using means and standard deviations. For non-normally distributed data, descriptive statistics will utilize median and percentile values (P25 and P75). Between-group comparisons will be carried out using t-tests, analysis of variance, chi-square tests, Mann-Whitney U tests, and statistical methods for repeated measures data to assess differences among the groups. The significance level in this study will be set at $\alpha=0.05$, with $P<0.05$ indicating statistical significance.

The changes in the primary outcome indicator VAS-P at different time points (0h, 1h, 6h, 12h, 24h, 48h, 72h) will be assessed by repeated measures analysis of variance (ANOVA) if the data conform to a normal distribution. Secondary outcome indicators, such as intra-abdominal pressure (IAP), will be analyzed in the same manner. The number of additional interventions (NAI) and the dose of coadministered medication (DCD) will be expressed as mean ± SD or median (IQR), and comparisons between groups will be made using the t-test or the Mann-Whitney test. The number of days of epidural placement (NDEP) will also be subjected to descriptive statistics and between-group comparisons. The number of cases of adverse events (AE) will be expressed as frequency, and comparisons between groups will be made using the chi-square test or Fisher's exact test. The outcome indicators, including puncture success rate (PSR), length of stay in the ICU (ICU-LOS), 28-day mortality rate (28D-MR), and satisfaction with analgesic effect score (SAE), will be subjected to descriptive statistics and between-group comparisons using the chi-square test or Fisher's exact test, as well as the t-test or the Mann-Whitney test.

In this study, faced with the problem of missing data, we would first try to obtain the missing data through retrospective collection. If this approach fails to work, we will take appropriate measures to deal with the problem depending on the nature of the missing data. For random missing data from consecutive repeated measurements, we can estimate this by calculating the average of the time points before and after the missing

data. If data are missing on two consecutive occasions, the patient's data are considered incomplete and the patient will be excluded. For data that are not missing at random, we will choose an appropriate interpolation method, such as the maximum deviation method or the Verbeke and Molenberghs method, on a case-by-case basis. When choosing interpolation methods, we will consider the distributional characteristics of the data and the missing mechanism to ensure the reliability and validity of the interpolation results.

25.Data management and monitoring

Data processing software such as GraphPad Prism, Excel, and Word will be utilized. Data collection and management will be conducted through Microsoft Excel by trained research assistants who are responsible for data entry and organization. All data will be regularly backed up and stored in encrypted form to ensure security. During analysis, statistical software will be used to process the data from Excel to evaluate the effects of the intervention.Data management and monitoring follows national and international standards of good clinical practice and complies with regulatory and ethical requirements. All relevant or irrelevant serious adverse events affecting patients will be collected and documented in source files. The quality of the study and regulatory compliance will be continuously monitored. Monitors will not be involved in the conduct of the study to ensure objectivity and independence. The Ethics Committee has approved the monitoring of the study to verify the qualifications of the investigators and study team members and to ensure the soundness and appropriateness of the documentation. In addition, data quality monitoring will be conducted through a variety of means to ensure accuracy, completeness and consistency of data.

26. Innovation:

This study is the first to explore the application of Symptom Management Theory (SMT) in epidural analgesia for patients with severe acute pancreatitis (SAP) led by nurses. Integrating SMT into pain management provides a structured approach for symptom assessment and intervention, which may enhance the effectiveness of pain control. The study also introduces a flexible analgesic regimen that combines continuous thoracic epidural analgesia (TEA) with intermittent injections on demand, aiming to offer more personalized and effective pain management. These innovative aspects may provide new insights for optimizing pain management plans for SAP patients and promote the role of nurses in pain management as well as the application of SMT in other clinical settings.

27. Limitations:

This study is a single-center trial, which may limit the generalizability of the study results. Although the sample size calculation ensures statistical power, it may still be insufficient to detect small but clinically significant

differences. Additionally, the study relies on the accurate assessment of pain using the Visual Analog Scale for Pain (VAS-P), which may be subject to patient subjective interpretation. There may also be data missing due to patient withdrawal or other unforeseen circumstances, which could affect the robustness of the results. Furthermore, the study duration may not be long enough to fully capture the long-term effects of the intervention.

28. Practical Significance:

Despite the limitations, this study holds significant practical value. By exploring the potential benefits of nurse-led pain management based on SMT, it paves the way for providing more personalized and effective pain management strategies for SAP patients. If the experimental group shows superior outcomes, this could lead to a shift in clinical practice, favoring intermittent epidural injections over continuous TEA. This would not only enhance patient comfort and satisfaction but also potentially reduce healthcare costs associated with prolonged ICU stays and adverse events. The study results may also encourage further exploration of the role of nurses in pain management and the application of SMT in other clinical settings.

29. Risks and Safety Measures of This Study

(1) Clear Notification of Reasonable Anticipated Risks or Inconveniences to Subjects:

In this study, subjects may face the following risks or inconveniences, including procedural risks (such as failed puncture, epidural hematoma, nerve injury, etc.), management risks (such as poor communication, coordination difficulties, non-standard data management, etc.), medication risks (such as incorrect drug dosage, drug allergic reactions, drug interactions, etc.), and poor patient compliance risks (patients may not cooperate with the study procedures or may drop out of the study midway due to pain, discomfort, or other reasons).

(2) Safety Measures to Be Taken in This Study:

Procedural Risk Management: The thoracic epidural puncture and catheter placement will be performed by experienced doctors to increase the success rate of puncture; medications will be used reasonably. Regular training and assessment of the puncture and medication administration procedures will be conducted for the medical staff involved in the study to ensure the proficiency and standardization of the operations; during the operation, the vital signs and reactions of the patients will be closely monitored, and the operation will be immediately stopped and corresponding measures taken if any abnormalities occur; a system for reporting adverse events will be established to promptly record and report adverse events related to the operation, and to conduct cause analysis and develop improvement measures.

Management Risk Management: An interdisciplinary research team will be established to clarify the responsibilities and tasks of each member, regular team meetings will be held to communicate and solve problems

that arise during the study in a timely manner; detailed research procedures and operating standards will be formulated to ensure that every link of the study is regulated and to reduce problems caused by chaotic management; a dedicated person will be assigned to manage the collection, entry, and management of data, with regular data backups and quality checks to ensure the integrity and accuracy of the data; a complete patient follow-up mechanism will be established to ensure that patients receive timely follow-up and necessary medical support during and after the study.

Medication Risk Management: Strict adherence to drug preparation and administration standards will be ensured to guarantee accurate drug dosages; clearly labeled drug containers will be used to avoid drug confusion; before using new drugs or drug combinations, patients' allergy histories will be inquired about and necessary allergy tests will be conducted, with antiallergic drugs and emergency equipment prepared to immediately handle any allergic reactions; during drug administration, the vital signs and reactions of patients will be closely monitored, and drug administration will be immediately stopped and corresponding measures taken if any abnormalities occur; training on drug knowledge and administration procedures will be provided to the medical staff involved in the study to ensure their familiarity with the pharmacological actions, adverse reactions, and handling methods of the drugs.

Poor Patient Compliance Risk Management: Before the start of the study, the purpose, methods, potential risks, and expected benefits of the study will be explained in detail to patients and their families to ensure their full understanding and signing of the informed consent form; during the study, regular communication with patients will be maintained to understand their feelings and needs and to answer questions in a timely manner; the pain management plan will be optimized to ensure effective pain relief for patients during the study, and the doses and administration methods of analgesic drugs will be adjusted in a timely manner based on patient feedback to improve patient comfort and compliance.

30. Rights, Benefits, and Compensation of Subjects Participating in This Study

(1) Informed Consent Acquisition:

The researchers will provide subjects with a detailed introduction to the research arrangements of the scientific research project. Participation is entirely voluntary, and researchers will report all events related to the subjects to enable them to decide at any time whether to continue participating.

(2) Benefits:

This study aims to compare the analgesic effects of different epidural medication methods on patients with severe acute pancreatitis (SAP), in order to provide new directions and theoretical support for the treatment of SAP patients, as well as to select the best epidural medication for SAP patients. This can help shorten the length of stay

in the ICU for patients, reduce medical costs, and alleviate the economic burden on patients.

(3) Compensation:None.

(4) Medical Care and Protection of Subjects in This Study:

This study will fully ensure the medical care and rights of subjects. Subjects may face the following risks:

Procedural Risks: Although epidural puncture and drug administration procedures are routinely performed in the department, there may still be procedural risks, such as failed puncture, epidural hematoma, nerve injury, etc. The sources of risk include procedural techniques, individual differences among patients, equipment failure, etc. The emergency plan includes personnel training, monitoring and handling, and reporting system.

Management Risks:The study involves interdisciplinary collaboration and complex management processes, and there may be problems such as poor communication, coordination difficulties, and non-standard data management. The sources of risk include interdisciplinary collaboration, data collection and management, patient follow-up, etc. The emergency plan includes establishing an interdisciplinary research team, optimizing processes, data management, and patient follow-up.

Medication Risks: Epidural drug administration involves the use of multiple drugs, and there may be risks such as incorrect drug dosage, drug allergic reactions, drug interactions, etc. The sources of risk include drug preparation, drug dosage, individual differences among patients, etc. The emergency plan includes drug management, allergy prevention, monitoring and handling, and drug training.

Poor Patient Compliance Risks: Patients may not cooperate with the study procedures or may drop out of the study midway due to pain, discomfort, or other reasons, which may affect the integrity of the study and the reliability of the results. The sources of risk include individual differences among patients, pain management effectiveness, and poor communication. The emergency plan includes communication and education, pain management.

32. Privacy and Confidentiality of Subjects

The storage, use, and confidentiality measures of subjects' personal information will be truthfully informed to the subjects, and their personal information will not be disclosed to third parties without authorization; there are confidentiality measures for subjects' personal information and related materials. This study has established a strict subject management system to ensure the rights and safety of subjects. All subjects are required to sign an informed consent form to fully understand the content of the study, potential risks, and the right to withdraw. During the study, subjects will be closely monitored by professional medical staff, and any discomfort or adverse reactions will be promptly handled and recorded. The research team will regularly communicate with subjects and their families to ensure their understanding of the study progress and participation.

33. Publication or Disclosure of This Study's Results

Regardless of the outcome, we will endeavor to publish the results of this study. Data management will follow strict standard operating procedures to ensure the accuracy, integrity, and confidentiality of the data. Data collection will be completed by an independent nurse data collector, and all data will be cross-verified by two researchers after collection to ensure accuracy before being entered into the system. All original data and records will be retained for five years after the study ends for subsequent audits and verification. Under the management of the ethics committee and the department, a data safety monitoring committee will be established for this study, composed of experts in statistics, clinical practice, and ethics. Its responsibilities include regularly reviewing the study progress, subject safety, and adverse events to ensure the scientific and compliant nature of the study. Meetings will be held once during the middle and once at the end of the study to review the data and provide suggestions.

34. Implementation Conditions and Researchers of This Study

(1) Whether the Study Site and Equipment Conditions Can Meet the Needs of the Study Tasks:

Study Site: Department of Critical Care Medicine, Affiliated Hospital of Zunyi Medical University.

Equipment Conditions:Ultrasound.

(2) Whether the Qualifications, Experience, and Technical Abilities of the Researchers Meet the Requirements of the Study:

This study relies on the Affiliated Hospital of Zunyi Medical University, which is a direct affiliated hospital of Zunyi Medical University, a university co-built by the National Health Commission (formerly the National Health and Family Planning Commission) and the People's Government of Guizhou Province. The predecessor of the hospital was the Affiliated Hospital of Dalian Medical University, which was relocated to Guizhou in 1969 to support the construction of the third line. It is the first tertiary general hospital in Guizhou Province and also the diagnosis and treatment center for severe acute pancreatitis in Guizhou Province. The department has anesthetists who have been engaged in critical care medicine for many years, which means that they have accumulated certain professional knowledge and skills in the relevant fields and can quickly adapt to and master new technologies, providing a solid technical basis for the smooth implementation of the project. The team has a reasonable personnel structure and rich clinical and research experience, which can provide a solid foundation for this study.

(3) Whether the Medical Practice Qualifications and Experience of the Researchers Can Ensure the Safety and Medical Care of the Subjects:

The research team consists of 14 members, including 2 project managers, 2 anesthesiologists, 3 clinical physicians,

5 pain management nurses, and 2 data collection researchers. Project managers are responsible for pain management training, nurse capability assessment, and study implementation supervision. Anesthesiologists are responsible for epidural catheter placement and analgesic plan adjustment. Clinical physicians manage the daily medical care of patients and participate in plan adjustments. Pain management nurses implement pain assessment and analgesia based on the symptom management theory, record pain scores and adverse reactions. Data collection researchers are responsible for data collection, organization, quality control, and analysis, using GraphPad Prism software to ensure the scientific nature of the results. Three members have hosted or participated in three provincial and municipal scientific research projects, published several papers, and applied for more than 10 patents. The team members have rich clinical work experience and are proficient in the diagnosis, treatment, and nursing processes of various SAP patients.

(4) Whether There Are Qualified Researchers or Researchers Trained to Obtain Informed Consent and Provide Consultation on Safety Issues at Any Time:

Dr. Fu Bao, one of the researchers in this project, has long been engaged in the clinical and research work of the Department of Critical Care Medicine. All members of the research team hold professional qualification certificates for physicians or nurses in their respective fields.

(5) Division of Labor Among Researchers:

Project Managers:Responsible for pain management training, nurse capability assessment, and study implementation supervision.

Anesthesiologists:Responsible for epidural catheter placement and analgesic plan adjustment.

Clinical Physicians: Manage the daily medical care of patients and participate in plan adjustments.

Pain Management Nurses: Implement pain assessment and analgesia based on the symptom management theory, record pain scores and adverse reactions.

Data Collection Researchers: Responsible for data collection, organization, quality control, and analysis, using GraphPad Prism software to ensure the scientific nature of the results.

35. SPIRIT-Figure

SPIRIT-Figure:Schedule of enrolment, interventions and assessments

	STUDY PERIOD												
	Enrolment	Allocation	Post-allocation										Close-out
**TIMEPOINT****	$-t_1$	0	t_1	t_2	t_3	t_4	t_5	t_6	t_7	t_8	t_x	t_y	t_z
**TIME**			*0h*	*1h*	*3h*	*6h*	*12h*	*24h*	*48h*	*72h*			
**ENROLMENT:**	X												
**Eligibility screen**	X												
**Review inclusion and exclusion criteria**	X												
**Informed consent**	X												
**Allocation**		X											
**Randomization Blinding**		X											
**INTERVENTIONS:**													
**[Control group]**			◆—	—	—	—	—	—	—	—	—◆		
**[Experimental group]**			◆—	—	—	—	—	—	—	—	—◆		
**ASSESSMENTS:**													
**Sociodemographic information and clinical status**	X	X											X
**Visual Analog Scale for Pain(VAS-P)**			X	X	X	X	X	X	X	X			
**Intra-abdominal pressure(IAP)**			X	X	X	X	X	X	X	X			
**Number of additional interventions(NAI)**								X	X	X			
**Dosage of combined drugs(DCD)**											X		
**Number of days of epidural placement(NDEP)**											X		
**Puncture-related adverse events Drug-related adverse events**			◆—	—	—	—	—	—	—	—	—◆		
**Puncture surgery rate (PSR)**			X										
**Length of stay in ICU (ICU-LOS)**												X	X
**28-day mortality rate (28D-MR)**												X	X
**Satisfaction score with analgesic effect (SAE)**										X			

t_x: Remove the thoracic epidural catheter

t_y and t_z: transfer out of the ICU or be admitted to the ICU for 28 days.

Figure 5:SPIRIT-Figure:Schedule of enrolment, interventions and assessments
